# The “Loci” of Misinformation and Its Correction in Peer- and Expert-Led Online Communities for Mental Health: Content Analysis

**DOI:** 10.2196/44656

**Published:** 2023-09-18

**Authors:** Nicole Bizzotto, Peter Johannes Schulz, Gert-Jan de Bruijn

**Affiliations:** 1 Faculty of Communication, Culture and Society Università della Svizzera italiana Lugano Switzerland; 2 Department of Communication and Media Ewha Womans University Seoul Republic of Korea; 3 Wee Kim Wee School of Communication & Information & LKC School of Medicine Nanyang Technological University Singapore; 4 Department of Communication Studies University of Antwerp Antwerp Belgium

**Keywords:** online communities, social media, mental health, misinformation, empowerment, content analysis, online community, infodemiology, information seeking, help seeking, information behavior, online search, search query, information quality, information accuracy

## Abstract

**Background:**

Mental health problems are recognized as a pressing public health issue, and an increasing number of individuals are turning to online communities for mental health to search for information and support. Although these virtual platforms have the potential to provide emotional support and access to anecdotal experiences, they can also present users with large amounts of potentially inaccurate information. Despite the importance of this issue, limited research has been conducted, especially on the differences that might emerge due to the type of content moderation of online communities: peer-led or expert-led.

**Objective:**

We aim to fill this gap by examining the prevalence, the communicative context, and the persistence of mental health misinformation on Facebook online communities for mental health, with a focus on understanding the mechanisms that enable effective correction of inaccurate information and differences between expert-led and peer-led groups.

**Methods:**

We conducted a content analysis of 1534 statements (from 144 threads) in 2 Italian-speaking Facebook groups.

**Results:**

The study found that an alarming number of comments (26.1%) contained medically inaccurate information. Furthermore, nearly 60% of the threads presented at least one misinformation statement without any correction attempt. Moderators were more likely to correct misinformation than members; however, they were not immune to posting content containing misinformation, which was an unexpected finding. Discussions about aspects of treatment (including side effects or treatment interruption) significantly increased the probability of encountering misinformation. Additionally, the study found that misinformation produced in the comments of a thread, rather than as the first post, had a lower probability of being corrected, particularly in peer-led communities.

**Conclusions:**

The high prevalence of misinformation in online communities, particularly when left uncorrected, underscores the importance of conducting additional research to identify effective mechanisms to prevent its spread. This is especially important given the study’s finding that misinformation tends to be more prevalent around specific “loci” of discussion that, once identified, can serve as a starting point to develop strategies for preventing and correcting misinformation within them.

## Introduction

### Background

Mental health is steadily becoming recognized as a public health problem [1], and individuals are increasingly turning to social network sites to seek information and support related to their symptoms and treatments [2-4]. This is because on these platforms they can find emotional support, anecdotal experiences, and a vast amount of information from like-minded individuals [3,4]. Recently, online communities for mental health symptoms (OCMHs) on social network sites have replaced previously used platforms, such as forums [2]. The use of OCMHs is not without pitfalls, as users are often presented with health-related information that may be of questionable validity or be contradictory [5-10]. Previous research has shown that OCMHs can lead to problematic self-diagnosis and self-treatment or exposure to harm-advocating or pro-anorexia content [11-14].

Additionally, online communities can foster the creation of echo chambers, where ideas are not challenged and users fall prey to confirmation bias and polarized viewpoints [15,16].

Research on misinformation related to mental health is scarce; previous research on the topic using methodologies such as content analysis has focused on physical illnesses or health in general, with little attention paid to mental health [7,17-21]. This is concerning, as people with mental health symptoms are also more vulnerable to the endorsement of misinformation [22,23], and the impact of mental health misinformation may have several negative consequences, ranging from reduced trust in health professionals to delayed or prevented effective care [7].

Previous research focused on understanding the factors that drive the spread of medical misinformation [16,24-28], with very few studies (eg, Ngai et al [17]) examining the context in which misinformation is present and the driving mechanisms.

Online health communities rely on the work of expert or peer volunteers to police themselves [29]. While some moderators are health professionals, others lack expert credentials [30-32]. Both can take actions such as deleting content or suspending users for inappropriate behavior.

Although health care professionals are crucial to ensuring the quality of information in online communities [33,34], and numerous studies have shown that expert content moderation can effectively help correct misinformation [35], limited research has been conducted on differences between different types of management of online health communities [36].

The present research focuses on a specific social network site: Facebook. Different sites may have different affordances, that is, unique features and functionalities that allow users to engage in specific types of social interactions [37]. Facebook’s question-and-answer format enables users to begin a thread asking a question (in the form of a post) and receive answers (as comments) from other members of the community.

Misinformation can be present in any part of this thread, and its specific location can influence its visibility; thus, it is possible for misinformation to be identified and corrected by other members or moderators.

There is a lack of research in this area and on the conditions that enable not only correction, but also effective correction, that is, correction that stops misinformation from being present in the subsequent comments in the thread.

Understanding the context in which mental health misinformation occurs, its driving mechanisms, and the factors contributing to its persistence is crucial for combating its spread. This study provides a novel perspective in that it concentrates not only on addressing misinformation prevalence but also on understanding the milieu of misinformation and related correction.

### Working Definition of Misinformation

Health misinformation is generally defined to as a “health-related claim of fact that is currently false due to a lack of scientific evidence” [16]. However, we sought to provide a new perspective by identifying and categorizing different types of misinformation emerging from an analysis of OCMH content, subsequently classified as content-related misinformation, context-related misinformation, wrong assumptions, and wrong terminology. Content-related misinformation refers to information that is false due to a lack of scientific evidence. Context-related misinformation refers to information that is not adequate for the context, either because the speaker lacks the necessary knowledge or the status to make certain inferences. We also introduce a specific category of misinformation in which the speaker asks for help based on incorrect assumptions. The last category is wrong terminology, which occurs when the speaker uses incorrect, stigmatizing, or inappropriate language. Examples are given for each category in the Methods section.

### Research Questions

The overall aim of this study is to investigate the prevalence, drivers, and characteristics of misinformation related to mental health in online communities and explore the factors that contribute to its correction or persistence (ie, when misinformation continues in the thread). Furthermore, we will explore whether these phenomena differ between peer-led and expert-led OCMHs.

More specifically, as we alluded to in the title by using the term *loci*, a Latin word that the Romans used to indicate places or positions within a larger context, we aim to investigate (1) the communicative environments where misinformation arises, and (2) whether it remains uncorrected (or not).

Based on the literature, we formulated the following subquestions to address the extent of the problem and examine the characteristics and contexts in which it occurs. Further details with respect to the specific variables included are provided in the “Operationalization of Variables” subsections.

- Research question 1: What is the prevalence of misinformation and of misinformation correction in OCMHs?
- Research question 2: What are the characteristics of users associated with spreading or correcting misinformation?
- Research question 3: What thread topics are associated with a higher prevalence of misinformation, and of which type? As previously mentioned, we will distinguish between different types of misinformation (ie, content, context, wrong assumptions, wrong terminology). Furthermore, we will investigate topics in the thread that are more associated with the presence of misinformation in terms of (1) type of illness, (2) the type of request made by the help seeker (informational or emotional support), and (3) the illness trajectory, that is, the stage at which the advice seeker needs informational or emotional help.
- Research question 4: What are the characteristics that might facilitate or impede misinformation correction, such as different types of content moderation (peer-led or expert-led) and the location of misinformation in the thread (first post or later comments)?

## Methods

This study approaches its aims using content analysis. The study protocol reports additional information on the sampling and methodology [30]. The coding was conducted by 2 expert coders (licensed psychologists with MSc degrees in psychology and health communication).

### Sampling Strategy

The content analysis was conducted on statements from 2 Italian Facebook OCMHs selected from among 14 Facebook groups that agreed to participate in the study. One of these was expert-led (with approximately 12,000 members) and the other was peer-led (with approximately 5500 members). Posts were randomly selected from each group so that for every month of the period from January 2019 to December 2021, 2 posts per group (peer-led or expert-led) were retrieved.

### Units of Analysis

Facebook groups use a question-and-answer format. A discussion starts with a post that is an individual message posted by one member. Later comments are responses to this post by other members of the group. Together, the post and its subsequent comments are called a “thread.” In this research, we focused only on Facebook comments posted at the first level, as according to the literature other levels easily contain parallel discussions [38].

We will refer from now on to individual messages posted in the OCMHs, either in form of posts or of comments, as statements. Statements will be the focus of some analyses, such as the prevalence of misinformation. For other types of analyses, we will use different categories of threads, such as those that contain or do not contain misinformation and related corrections (see the following section “Veracity of Threads”). Furthermore, we differentiated whether statements were requests for or provisions of help.

### Operationalization of Variables

#### Veracity of Threads

To address the prevalence of misinformation and of misinformation correction at the level of threads, we computed a new variable called *veracity of threads*.

Following Nyhan and Reifler [39], we distinguished 3 different types of categories: (1) threads without misinformation, (2) threads with corrected misinformation (“corrected”), and (3) threads with uncorrected misinformation (“uncorrected”).

#### Characteristics of Users

We will consider variables such as gender, group status (member, moderator), type of advice given (emotional help, declarative knowledge, procedural advice, or call to action, ie, referral to a health professional). Declarative knowledge is a type of knowledge that can be verbalized and taught on that basis (eg, the symptoms of a panic attack). Procedural knowledge, on the other hand, pertains to an individual’s understanding of how something operates (eg, how to manage medications).

#### Typology of Misinformation

Misinformation was categorized into content-related misinformation, context-related misinformation, wrong assumptions, or wrong terminology. An example of the first category (content misinformation) would be a comment suggesting herbal cures for psychiatric symptoms. Herbal medicine in most cases is not immediately harmful but may delay effective professional help seeking. An example of the second category (context misinformation) would involve a member of an OCMH suggesting to a peer that they may be experiencing symptoms of an anxiety disorder. Furthermore, a category specific to misinformation was added to classify when the questioner asks for help while basing the request on wrong assumptions (eg, “antidepressants do not work for me, can you recommend a natural method?”). The last category considered in the study is wrong terminology, a milder type of misinformation that occurs when statements contain incorrect, stigmatizing, or otherwise inappropriate terminology. Referring to depressed patients as “lazy crazy people” is an example. In the analyses including misinformation typologies, the wrong terminology category was dropped, as the observed frequencies were less than 5. The category of wrong assumptions is important because it pertains to misinformation that originates from the initial post by the help seeker, as opposed to misinformation that arises later in the comments. This is crucial for interpreting findings related to misinformation correction, given that the visibility of the misinformation might vary based on when it is introduced; in the comments section, it might be less noticeable and more difficult to detect.

#### Thread Topics

We investigated (1) types of illness (we focused on the 3 most prevalent: mood disorders, anxiety disorders, and physical symptoms), (2) types of request by the help seeker (coded as requests for declarative knowledge, procedural knowledge, or emotional support), and (3) the trajectory of the illness, that is, the stage at which the advice seeker needs informational or emotional support (related to causes, diagnosis, symptoms, and treatment) with a specific attention to the different typologies of treatment options (psychotherapy, medication, and complementary or alternative medicine). There were more categories in our initial study protocol [30], but we decided to aggregate them for the sake of clarity.

### Ethical Considerations

This research has been approved by the Università della Svizzera italiana (CE_2021_4). The guidelines outlined in previous social media research informed this study’s procedures [40], including irreversibly anonymizing the data and removing any other personal information that could potentially result in a breach of anonymity or privacy and reveal any information that could be attributed to a single individual (eg, photographs, locations). Consent to analyze the posts was required by the Facebook communities’ owners (administrators) prior to data collection.

### Analysis

As the units of analysis mainly consisted of categorical data, Pearson chi-square tests were conducted separately. A *P* value of less than .05 was considered statistically significant. Cramér V or φ were used to identify the effect size [41].

## Results

### Descriptive Statistics

The sample included 1534 units of analysis (144 threads) generated by 1037 members of the OCMHs. The average number of comments were distributed in the 3 years as follows: 18.73 (SD 2.07) comments in 2019, 25.50 (SD 3.18) comments in 2020, and 22.40 (SD 2.49) comments in 2021. As a result, in our analysis, the number of comments analyzed per thread was on average 9.67 (median 8.00; SD 8.51; range 1-66).

From the available information, 71.6% (742/1037) of statements were posted by women. The most common type of illness addressed was anxiety disorders, which were present in 59.7% (916/1534) of statements. The second most common was depression, appearing in 35.7% (548/1534) of statements. Many posts addressed both these illnesses (377/1534, 25%).

The third most common illness was related to aspects related to physical symptoms (435/1534, 28.4%). This topic was also frequently mentioned together with anxiety (219/1534, 14.3%). Suicide-related conversations were also highly prevalent in the discussions (156/1534, 10.2%). The following results are organized by research questions.

### Research Question 1: What Is the Prevalence of Misinformation and Misinformation Correction?

Of the 144 threads analyzed, which contained 1534 statements, 1390 were comments (1534–144=1390). Of the 144 threads, a little more than one-fifth (n=31, 21.5%) contained no misinformation, one-fifth (n=29, 20.1%) contained statements with misinformation but with correction, and nearly 60% (n=84, 58.3%) contained misinformation that was not corrected. At the level of statements, 401/1534 (26.1%) contained misinformation.

### Research Question 2: What Are the Characteristics Associated With Spreading or Correction of Misinformation?

#### Gender Differences

Although men posted and commented less (n=436) than women (n=1098), there were no significant gender differences in statements containing misinformation (28.9% for men, 25% for women; *χ*^2^_1_=2.40; *P*=.12). There were also no gender differences in correcting misinformation (28/436, 6.4% for men, 55/1098, 5% for women; *χ*^2^_1_=1.22; *P*=.27).

#### Group Status

Moderators made a total of 61 statements, whereas members made a total of 1473 statements. Of the statements from the moderators, 15/61 (24.6%) contained misinformation, whereas members made a total of 386 statements that contained misinformation (386/1473, 26.2%); this difference was not significant (*χ*^2^_1_=0.79; *P*=.78). Moderators made a total of 11 corrections (11/61,18%) and members a total of 72 corrections (72/1473, 4.9%), which was significantly different (*χ*^2^_1_=19.77; *P*<.001) with a small effect size (φ=0.11).

#### Advice Type

Of the 1534 statements, 1182 contained advice on a previous post. These 1182 statements were divided into four types of communication: (1) declarative knowledge, (2) procedural knowledge, (3) calls to action, and (4) emotional support.

The majority of these posts were related to emotional support (413/1182, 34.9%), followed by advice on procedural knowledge (333/1182, 28.2%) and declarative knowledge (269/1182, 22.8%). Calls to action were mentioned fewest (167/1182, 14.1%). These different types of advice contained significantly different rates of misinformation (*χ*^2^_3_=287.605; *P*<.001; V=.493): 56.6% (152/269) for declarative knowledge, 44.7% (149/333) for procedural advice, and 9.2% (38/413) for emotional support. Call to action posts did not contain misinformation.

### Research Question 3: Topics and Types of Misinformation

As mentioned above, 401 statements contained misinformation. Among these, the most prevalent type of misinformation was context misinformation (225/401, 56.1%), followed by content misinformation (118/401, 29.4%), wrong assumptions (53/401, 13.2%), and wrong terminology (5/401, 1.2%).

Table 1 reports the numbers and proportions of statements containing misinformation for the 6 topics we coded. There were no significant differences in misinformation proportion for type of illness (*P*=.65) or motivation to seek help (*P*=.35). However, there were significant differences in misinformation proportions when topics related to treatments were discussed (for typology, treatment interruption, and adverse effects) or when illness trajectories were discussed.

**Table 1:** Prevalence of misinformation in different topics of discussion.

Topic and levels (number of statements)		Statements containing misinformation, n (%)	Chi square (*df)*		*P* value	
**Type of illness (n=1004)^a^**				.870 (2)		.66
	Mood disorders (n=242)	64 (26.4)				
	Anxiety disorders (n=563)	145 (25.8)				
	Physical symptoms (n=199)	58 (29.1)				
**Trajectory of illness (n=1534)**				63.697 (4)		.001^b^
	Causes (n=91)	54 (59.3)				
	Treatment options (n=276)	83 (30.1)				
	Diagnosis regarding symptoms (n=378)	85 (22.5)				
	Treatment for specific symptoms (n=365)	74 (20.3)				
	Treatment effectiveness (n=424)	105 (24.8)				
**Typology of treatments (n=552)^c^**				67.07 (2)		.001^d^
	Psychotherapy (n=155)	22 (19.1)				
	Psychotropic medications (n=281)	73 (26)				
	Complementary and alternative medicine (n=156)	94 (60.3)				
**Motivation to seek help (n=168)^e^**				2.098 (2)		.35
	Declarative knowledge (n=48)	17 (35.4)				
	Procedural knowledge (n=57)	19 (33.3)				
	Emotional support (n=63)	15 (23.8)				
**Treatment interruption (n=700^f^)**				7.69 (1)		.006^g^
	Not mentioned in the statement (n=635)	187 (29.4)				
	Mentioned in the statement (n=65)	30 (46.2)				
**Adverse effects of treatment (n=700^f^)**				4.43 (1)		.04^h^
	Not mentioned in the statement (n=620)	184 (29.7)				
	Mentioned in the statement (n=80)	33 (41.3)				

^a^All statements were referring to a specific type of illness (see study protocol for a complete list). However, for the sake of simplicity and clarity, we aggregated different types of illnesses into mood disorders, anxiety disorders, and physical symptoms. Statements containing references to more than one type of illness were excluded from the analysis.

^b^V=0.20.

^c^Statements discussing more than one treatment topic were dropped from the analysis for the sake of clarity.

^d^V=0.35.

^e^Misinformation here is only of the wrong assumptions type.

^f^Statements discussing treatment.

^g^φ=0.10.

^h^φ=0.08.

### Research Question 4: What Are Characteristics That Facilitate or Impede Misinformation Correction?

#### Type of Content Moderation

Of the 692 statements retrieved from the peer-led OCMH, 224 (32.4%) contained misinformation, while in the expert-led OCMH, 177 statements of 842 contained misinformation (21%), which was a significant difference (*χ*^2^_1_=25.337; *P*<.001) with a small effect size (φ=0.13).

With regards to the correction of misinformation, our analyses focused on the level of threads. Of a total of 144 threads, 113 (78.5%) contained misinformation. Of these 113 threads, 60 (53.1%) were in the peer-led OCMH and 53 (46.9%) were in the expert-led OCMH. Of the 113 threads, in 29 (25.7%) misinformation was corrected: 10 of 29 (34.5%) in the peer-led OCMH and 19 of 29 (65.5%) in the expert-led OCMH, a significant difference (*χ*^2^_1_=5.428; *P*=.02) with a medium effect size (φ=0.22).

Regarding the opening post in the 113 threads that contained misinformation (of the type “wrong assumption”), 28 of 60 (46.7%) in the peer-led OCMH started with misinformation, whereas 17 of 53 (32.1%) in the expert-led OCMH started with misinformation; however, this difference was not significant (*χ*^2^_1_=2.500; *P*=.11). When looking at whether or not misinformation was later corrected, results showed that 9 of 28 (32.1%) posts with misinformation were corrected in the peer-led group, whereas 11 of 17 (64.7%) of the posts were corrected in the expert-led group. This difference was significant, (*χ*^2^_1_=13.83 *P*<.001), with a medium effect size (φ=0.35).

## Discussion

### Principal Findings

This study aimed to contribute to knowledge on the “loci” of misinformation and its correction. The results for research question 1 revealed that misinformation was highly prevalent, which was particularly concerning, as nearly 60% of the threads contained misinformation without any correction attempt.

Regarding research question 2, no gender differences emerged with respect to posting or correcting misinformation. Proportionwise, moderators posted the same amount of misinformation as other members. However, they were more likely to correct misinformation. Furthermore, misinformation tended to be more prevalent when the advice giver provided information (either procedural or declarative) rather than offering emotional support.

Regarding research question 3, the specific type of illness that was discussed did not have a significant impact on the prevalence of misinformation. However, the topic of treatment, particularly related to complementary and alternative medicine or treatment options, adverse effects of medication, and treatment interruption, was associated with a higher likelihood of misinformation.

Regarding research question 4, misinformation prevalence was significantly different in peer- and expert-led OCMHs (32.4% and 21%, respectively). Furthermore, when a thread started with a first post containing misinformation, it had a higher chance of being corrected in expert-led OCMHs. This is despite the finding that the occurrence of misinformation in the first post was not different from the peer-led OCMHs.

The results of this study, which to the best of our knowledge is the first to conduct a content analysis on mental health misinformation in online communities, highlight the importance of investigating aspects of online communities that could prevent the spread of inaccurate information that could potentially influence the beliefs and attitudes of OCMH users, including those related to formal help seeking.

Treatment was one of the most discussed stages in the illness trajectory, as was found in a similar study [42]. However, these discussions also had one of the highest rates of misinformation overall. Rates of misinformation in general were comparable to those found in other studies in different fields [43]. The absence of gender differences in the sharing of misinformation is also in line with other studies [44,45]. Furthermore, results showed that the location of misinformation in the thread was important: misinformation in the first post of a thread had a higher chance of being corrected than misinformation later in the thread, particularly in the expert-led OCMH. One possible explanation for this is that misinformation in the initial post might be more visible to other group members and moderators, thus increasing the likelihood of correction.

Previous studies also support the necessity of mental health experts being present in these communities [46,47]. However, a surprising finding was the fact that moderators were as likely as other members of the OCMHs to share misinformation. This aspect should be investigated in further studies.

### Limitations

Two main limitations of this work are worth mentioning. First, we used a limited number of units of analysis in the OCMHs (2 threads per month, when the average number of threads was approximately 900 and 600 for the larger and smaller groups analyzed, respectively). This was mainly due to the methodology used (human coding). Second, it is worth noting that as moderators have the ability to censor comments in their OCMHs, such corrections may not have been captured by our methodology.

### Conclusion

This study analyzed peer- and expert-led OCMHs to understand the prevalence of misinformation and related correction. The study found that an alarming number of comments contained medically inaccurate information or challenged medical expertise, especially around specific loci of discussion.

This study highlights the importance of having mental health experts present in these communities; given the significant amount of misinformation, it is crucial to implement greater control and censorship of information shared in OCMHs.

The study also found that correction of misinformation was more effective when it was more visible; thus, the integration of artificial intelligence in content moderation could assist administrators in detecting and correcting misinformation. This should especially be implemented for specific loci that were found to be more prone to contain misinformation, such as treatment options. In addition, this is the first study to categorize different types of mental health misinformation that proved relevant and should be considered for further investigation.

Future studies should address the impact of exposure to varying amounts of misinformation while taking into account individual differences, such as health literacy or patient empowerment, and differences related to the type of OCMH participation (ie, moderated by experts vs peers).

## Abbreviations

OCMH: online community for mental health

## Data Availability

The data that support the findings of this study are available from the corresponding author upon reasonable request.
